# Soluble Guanylate Cyclase Stimulator, BAY41‐8543: A Promising Approach for the Treatment of Chronic Heart Failure Caused by Pressure and Volume Overload

**DOI:** 10.1002/prp2.70087

**Published:** 2025-03-30

**Authors:** Adriana Martišková, Matúš Sýkora, Natália Andelová, Miroslav Ferko, Olga Gawrys, Katarína Andelová, Petr Kala, Luděk Červenka, Barbara Szeiffová Bačová

**Affiliations:** ^1^ Centre of Experimental Medicine Slovak Academy of Sciences, Institute for Heart Research Bratislava Slovakia; ^2^ Centre for Experimental Medicine Institute for Clinical and Experimental Medicine Prague Czech Republic

**Keywords:** heart failure with reduced ejection fraction, reactive oxygen/nitrogen species, renin‐angiotensin system, soluble guanylate cyclase stimulator

## Abstract

Heart failure (HF) is a leading cause of morbidity and mortality, often driven by prolonged exposure to pathological stimuli such as pressure and volume overload. These factors contribute to excessive oxidative stress, adverse cardiac remodeling, and dysregulation of the nitric oxide‐soluble guanylate cyclase‐cyclic guanosine monophosphate (NO‐sGC‐cGMP) signaling pathway. Given the urgent need for effective treatments, this study investigated the potential of sGC stimulators to mitigate HF progression. We utilized male hypertensive Ren‐2 transgenic (TGR) rats and a volume‐overload HF model induced by an aortocaval fistula (ACF). Rats received the sGC stimulator BAY 41–8543 (3 mg/kg/day) for 30 weeks, while normotensive Hannover Sprague–Dawley rats served as controls. At the study endpoint (40 weeks of age), left ventricular tissue was analyzed using mass spectrometry, Western blotting, and histological assessment. TGR rats treated with sGC stimulators exhibited a significant increase in key antioxidant proteins (SOD1, CH10, ACSF2, NDUS1, DHE3, GSTM2, and PCCA), suggesting enhanced resistance to oxidative stress. However, sGC stimulator treatment also upregulated extracellular matrix remodeling markers (MMP‐2, TGF‐β, and SMAD2/3), which are typically associated with fibrosis. Despite this, Masson's trichrome staining revealed reduced collagen deposition in both TGR and TGR‐ACF rats receiving sGC stimulators. Notably, all untreated TGR‐ACF rats succumbed before the study endpoint, preventing direct assessment of sGC stimulator effects in advanced HF. These findings highlight the therapeutic potential of sGC stimulators in HF, particularly through their antioxidant effects. However, their concurrent influence on fibrosis warrants further investigation to optimize treatment strategies.

## Graphical abstract

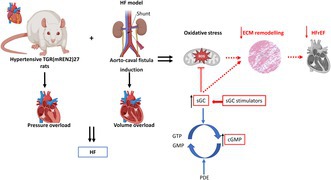

**Abbreviations**
ACEi: Angiotensin‐converting enzyme inhibitor
ACF: Aortocaval fistula
cGMP: Cyclic guanosine monophosphate
ECM: Extracellular matrix
HanSD: Normotensive Hannover Sprague–Dawley rats
HF: Heart failure
NO: Nitric oxide
RAAS: Renin‐angiotensin system
rEF: Reduced ejection fraction
ROS/RNS: Reactive Oxygen/Nitrogen Species
sGC: Soluble guanylate cyclase
TGR: Hypertensive Ren‐2 transgenic rats

## 1 Introduction

Heart failure (HF) is a serious health problem affecting millions of people worldwide, with a negative increasing impact on morbidity and mortality. The potential risk of developing HF has risen to 24%, meaning that approximately one in four people will develop HF during their lifetime [1].

HF can arise from various causes, including functional or structural impairment, through multiple mechanisms, and is often developed as a result of underlying cardiovascular diseases or conditions that weaken the heart's ability to pump or receive blood to ensure the metabolic needs of tissues [2, 3].

A crucial role in HF development plays the endothelial dysfunction. This dysfunction leads to a reduction in the production of nitric oxide (NO), degradation of endothelial nitric oxide synthases, and elevated formation of peroxynitrite, resulting in pro‐oxidant activity and a pro‐inflammatory state [4, 5]. This process is closely associated with reduced production of cyclic guanosine monophosphate (cGMP), which triggers a wide range of pathophysiological processes, including hypertrophy, cardiac fibrosis, and impaired contractility [6, 7, 8].

Low NO bioavailability and reduced cGMP are significantly present in patients with HF, for whom current treatment strategies are still poorly effective. Therefore, the NO‐cGMP pathway represents an important target in the treatment of HF [9]. One possible approach to targeting the NO‐cGMP pathway is through the use of soluble guanylate cyclase (sGC) stimulators and activators, which upregulate the enzymatic activity of sGC, catalyzing the synthesis of cGMP [10]. Besides the main role of sGC stimulators to maximize cGMP production, the ability to neutralize damage from oxidative stress was also demonstrated [11, 12].

**FIGURE 1:**
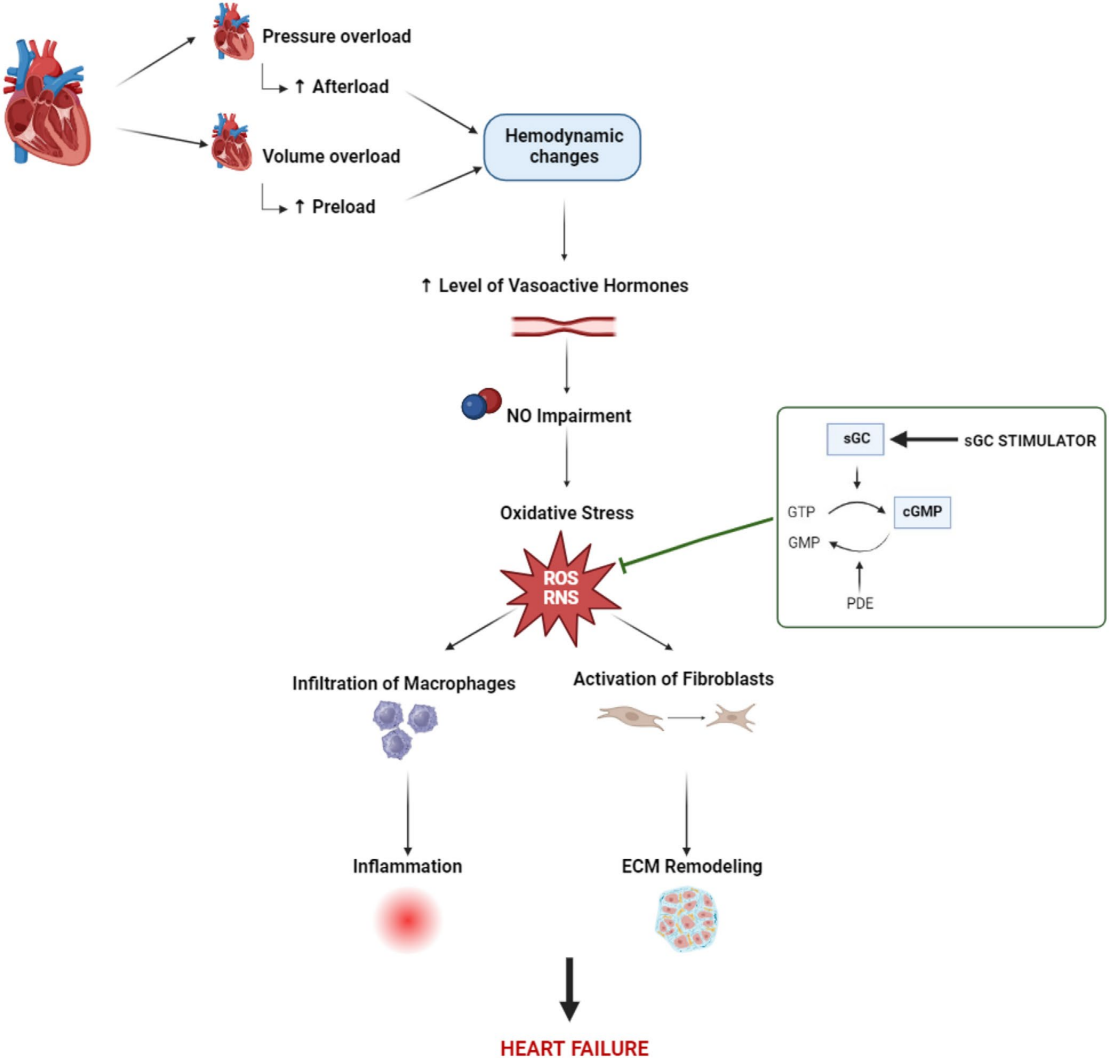
Schematic representation of possible cardioprotective action of sGC stimulator. Pressure and volume overload result in ventricular wall tension leading to hemodynamic changes, endothelial dysfunction, and oxidative damage. In addition, prolonged elevated levels of vasoactive hormones and an excess of free radicals can activate fibroblast‐mediated extracellular matrix remodeling as well as macrophage infiltration‐induced inflammatory responses. The sGC stimulator as a possible antioxidant can reduce oxidative stress and its consequences, which can lead to the improvement of heart failure. cGMP: Cyclic guanosine monophosphate; ECM: Extracellular matrix; NO: Nitric oxide; ROS/RNS: Reactive Oxygen/Nitrogen Species; sGC: Soluble Guanylate Cyclase (Created in BioRender.com).

Since oxidative stress is known to induce changes in cardiac remodeling through the development of apoptosis, necrosis, and fibrosis [13, 14], we hypothesized that treating rats with HF using the sGC stimulator (BAY41‐8543) could alleviate significant structural remodeling of the heart and, thus, at least partially contribute to the improvement of the alarmingly low survival rate (Figure 1).

To test this hypothesis, we used hypertensive, heterozygous Ren‐2 transgenic rats (TGR) with surgically created aortocaval fistula (ACF), which represent a model of high‐output HF [15, 16]. Long‐term treatment (210 days) with the sGC stimulator (BAY41‐8543) was performed, providing sufficient time to observe significant structural changes in the heart that were developed/reversed, influenced by the therapy or the underlying disease process, especially in the context of chronic conditions such as HF.

## 2 Methods

### 2.1 Experimental Animals

Experimental animals used in the present study were obtained from stock animals supplied by the Max Delbrück Center for Molecular Medicine (Berlin, Germany) and were bred at the Center of Experimental Medicine of the Institute for Clinical and Experimental Medicine (Prague, Czech Republic). The animals were kept on a 12‐h/12‐h light/dark cycle and had access to tap water ad libitum throughout the whole observation.

Male TGR [strain name TGR(mRen2)27] harboring the mouse Ren‐2 renin gene, at the initial age of 8 weeks, was used for experiments. At this stage, TGR rats are already in the sustained phase of hypertension, exhibiting systolic blood pressure levels comparable to those of hypertensive patients (i.e., systolic BP around 200 mmHg), along with significant activation of the endogenous renin‐angiotensin system (RAAS) [17]. Normotensive Hannover Sprague Dawley (HanSD) rats were used as a control.

The study was performed in accordance with the guidelines and practices established by the Animal Care and Use Committee of the Institute for Clinical and Experimental Medicine (Prague), approved by the Ministry of Health of the Czech Republic (decision number MZDR 12482/2021‐5/OVZ), and in compliance with the European Union Directive 63/2010. The study was carried out in compliance with the ARRIVE (Animals in Research: Reporting In vivo Experiments) guidelines [18].

### 2.2 Experimental Design

Chronic HF due to volume overload was induced by creating a fistula between the vena cava and the abdominal aorta [15, 19]. Rats were anesthetized before this procedure with an intraperitoneal injection of a ketamine/midazolam mixture (Calypsol, Gedeon Richter, Hungary, 160 mg/kg and Dormicum, Roche, France, 160 mg/kg). Sham‐operated rats underwent the same operation without creating the ACF.

Two weeks after the ACF or sham operation, rats were treated with the sGC stimulator (BAY41‐8543, 3 mg/kg/day) and an angiotensin‐converting enzyme inhibitor (ACEi, Trandolapril, 0.25 mg/kg/day). The dosage of the sGC stimulator was set according to Gawrys et al., 2023 [20].

Based on the treatment, rats were divided into the following experimental groups:

1. Sham‐operated HanSD rats without treatment (HanSD sham), *n* = 9;
2. Sham‐operated TGR rats without treatment (TGR sham), *n* = 9;
3. Sham‐operated TGR rats treated with the sGC stimulator BAY41‐8543 (TGR sGC), *n* = 10;
4. TGR rats with ACF without treatment (TGR ACF), *n* = 25;
5. TGR rats with ACF treated with sGC stimulator BAY41‐8543 (TGR ACF sGC), *n* = 28;
6. TGR rats with ACF treated with the inhibitor ACE trandolapril (TGR ACF ACEi), *n* = 24;
7. TGR rats with ACF treated with a combination of drugs (TGR ACF ACEi + sGC), *n* = 27.

The observation period lasted for 210 days. Afterwards, the animals were decapitated, and blood plasma and hearts were collected for further analyses.

### 2.3 Nano‐Liquid Chromatography and Mass Spectrometry‐Based Proteomics

Proteomic analysis of frozen tissue homogenates from the left ventricle of the heart was performed using a Nano System liquid chromatograph (Ultimate 3000 RSLC, Thermo Fisher Scientific, Germering, Germany) followed by mass spectrometry (LC–MS) with electrospray ionization (ESI) and a 3D ion trap mass analyzer (Amazon SL, Bruker, Bremen, Germany). Samples containing 250 μg of protein were digested with trypsin in solution (Sigma–Aldrich, USA) at a ratio of 1:25 (trypsin concentration: 0.2 μg/μL) overnight at 37°C. Prior to LC–MS proteomic analysis, the samples were desalted using C18‐U SPE columns (Strata, Phenomenex). This sample preparation procedure was performed as described by Andelova et al. [21]. For LC–MS analysis, the mobile phase consisted of component A (0.1% formic acid in 2% acetonitrile (ACN)) and component B (0.1% formic acid in 95% ACN). The loading phase consisted of 0.05% formic acid in 2% ACN. The mobile phase flow rate was 0.4 μL/min with a gradient length of 290 min: 4%–30% B for 258 min, followed by 12 min at 95% B and 20 min at 4% B. The spectrometer parameters were configured as follows: capillary voltage, 1450 V; atomizer pressure, 0.4 Ψ; gas flow rate, 3.0 L/min; gas temperature, 150°C; positive polarity; and a scan range of 50–2200 m/z with a scan rate of 8100 m/z/s. Additional LC–MS parameters, as well as the database searching and protein identification methods, were previously described [21].

### 2.4 Western Blot Analysis

As was described in our previous studies [22, 23], frozen tissue from the left ventricle of the heart was homogenized in lysis buffer [50 mmol/L Tris–HCl, 250 sucrose, 1.0 mmol/L EGTA, 1.0 mmol/L dithiothreitol, 1.0 mmol/L phenylmethylsulfonyl fluoride and 0.5 sodium orthovanadate (pH 7.4)] and diluted in the Laemmli sample buffer. Protease inhibitor cocktail was used as well (Sigma‐Aldrich, St. Louis, MO, USA, #P8340). Loading samples were then separated in 10% SDS‐PAGE acrylamide gels at a constant voltage of 90 V (Mini‐Protean TetraCell, Bio‐Rad, Hercules, CA, USA), electrically transferred to the nitrocellulose membrane (0.2 μm pore size, Advantec, Tokyo, Japan), and subsequently blocked with 5% skim milk. Membranes were incubated overnight in primary antibodies, washed in TBS‐T, and incubated in secondary antibodies for 1,5 h (Table 1).

**TABLE 1:** Primary and secondary antibodies used for Western blot analysis.

Antibody	Host	Dilution	Catalog no.	Supplier
Anti‐galectin‐3	Rabbit	/1:1000/	#89572	Cell Signaling Technology, Danvers, MA, USA
Anti‐GAPDH	Rabbit	/1:1000/	sc‐25 778	Santa Cruz Biotechnology, Texas, USA
Anti‐pkcδ	Rabbit	/1:1000/	sc‐213	Santa Cruz Biotechnology, Texas, USA
Anti‐MMP2	Rabbit	/1:500/	sc‐10 736	Santa Cruz Biotechnology, Texas, USA
Anti‐nfκb	Rabbit	/1:500/	sc‐372	Santa Cruz Biotechnology, Texas, USA
Anti‐PKG	Rabbit	/1:1000/	sc‐25 429	Santa Cruz Biotechnology, Texas, USA
Anti‐SMAD2/3	Rabbit	/1:1000/	#3102	Cell Signaling Technology, Colorado, USA
Anti‐TGF‐β1	Rabbit	/1:1000/	SAB4502954	Sigma‐Aldrich, Missouri, USA
Anti‐SOD1	Rabbit	/1:500/	sc‐11 407	Santa Cruz Biotechnology, Texas, USA
Anti‐Keap1	Mouse	/1:500/	sc‐514 914	Santa Cruz Biotechnology, Texas, USA
Anti‐Nrf2	Mouse	/1:500/	sc‐365 949	Santa Cruz Biotechnology, Texas, USA
Anti‐MPO	Mouse	/1:500/	sc‐52 707	Santa Cruz Biotechnology, Texas, USA
Anti‐HSP70	Mouse	/1:500/	sc‐32 239	Santa Cruz Biotechnology, Texas, USA
Anti‐Mouse	—	/1:2000/	#7076S	Cell Signaling Technology, Danvers, MA, USA
Anti‐Rabbit	—	/1:2000/	#7074S	Cell Signaling Technology, Danvers, MA, USA

Proteins were visualized by the enhanced luminol‐based chemiluminescence method. For a densitometric analysis of observed proteins, normalized to GAPDH, Carestream Molecular Imaging Software (version 5.0, Carestream Health, New Haven, CT, USA) was used.

### 2.5 Histological Masson's Trichrome Staining

For collagen detection by Masson's trichrome staining, 10 μm thick cryosections from the left ventricular tissue were fixed in Bouin's solution Mordant for 1 h at a temperature of 60°C. The sections were subsequently stained in Weigert's iron hematoxylin in a 1:1 ratio for 5 min, Biebrich Scarlet‐Acid Fuchsin for 15 min, and differentiated in Phosphomolybdic‐phosphotungstic acid solution for 10 min. The next step was staining in Aniline Blue solution for 5 min. Cryosections were then dehydrated very quickly through 95% ethyl alcohol, absolute ethyl alcohol, and cleared in xylene, poured with Canada balsam, and covered with a coverslip. Between individual steps, the sections were washed in running tap water or distilled water.

The stained areas were observed and captured by light microscope (Zeiss Apotome 2 microscope Carl Zeiss, Jena, Germany). Collagen fibers stain blue, nuclei stain black, and cytosol, keratin, and muscle fibers stain red.

For quantification of collagen deposition, 15 randomly selected areas of positive signal per sample were analyzed. We initially performed deconvolution of all images to the blue color component (channel), followed by conversion of the RGB‐format images to 8‐bit grayscale. Collagen deposition was measured as the area of pixels with a value below 128 on the 8‐bit grayscale, which ranges from black (0) to white (255), using Image‐Pro Plus (Media Cybernetics, Rockville, MD, USA) [24].

### 2.6 Histochemical Reaction of β‐Hydroxybutyrate Dehydrogenase

The enzyme β‐hydroxybutyrate dehydrogenase catalyzes the conversion of acetoacetate and β‐hydroxybutyrate, coupled with NAD^+^/NADH. Both of these compounds belong to ketone bodies, which act as the suppliers of energy in conditions of impaired glucose or oxygen delivery to tissues, which is typical also for advanced heart failure [25].

Enzyme histochemical reaction for the detection of the activity of β‐hydroxybutyrate dehydrogenase was performed according to Lojda et al. (1976) [26] on 10 μm thick cryosections from the left ventricular tissue. The cryosections were incubated for 20 min at 37°C in the Nicotinamide adenine dinucleotide with β‐hydroxybutyrate sodium incubation solution. After incubation, the sections were rinsed 3 times in distilled water and then fixed for 5 min in 4% formaldehyde solution. The sections were then embedded in gelatin. Histochemical reaction areas were observed and captured by light microscope (Zeiss Apotome 2 microscope Carl Zeiss, Jena, Germany). Intensity of staining correlates with the enzymatic activity of β‐hydroxybutyrate dehydrogenase.

For quantification of β ‐hydroxybutyrate dehydrogenase enzyme activity, 15 randomly selected areas per sample were analyzed. Microscopic images (RGB format) were converted to 8‐bit grayscale. Positive signal was defined as the area of pixels with a code lower than 135 on the 8‐bit grayscale, which ranges from black (0) to white (255), and the analysis was performed using Image‐Pro Plus (Media Cybernetics, Rockville, MD, USA).

### 2.7 Statistical Analysis

The GraphPad Prism 8.0.1 (GraphPad Software Inc., USA) was used for the statistical analysis of the obtained data, as well as their graphical display. Data were evaluated by 1‐way analysis of variance (ANOVA) followed by Tukey's post hoc test to compare statistically significant differences between groups.

Data were expressed as means ± standard deviations (SD); *p* < 0.05 was considered statistically significant.

LC–MS proteomic analyses were performed on three experimental groups (HanSD, TGR, and TGR sGC). Proteins identified in the intersection of all biological and technical replicates were quantified. This study focused on proteins primarily involved in structural remodeling and oxidative damage/antioxidant defense. Protein abundances were evaluated using the exponentially modified Protein Abundance Index (emPAI). The retrieved emPAI values were further used to calculate the “fold change” (FC), defined as the ratio of protein abundances under two different experimental conditions, averaged across replicates for each condition. To assess differences in protein abundance levels between experimental conditions, a *t* test was performed on log‐transformed emPAI data. Additionally, a two‐way ANOVA was used to analyze data from factorial experiments, with the sustained phase of hypertension (TGR) as the first main factor and treatment with the sGC stimulator as the second main factor.

## 3 Nomenclature of Targets and Ligands

Key protein targets and ligands in this article are hyperlinked to corresponding entries in http://www.guidetopharmacology.org, the common portal for data from the IUPHAR/BPS Guide to PHARMACOLOGY [26], and are permanently archived in the Concise Guide to PHARMACOLOGY 2019/20 [27].

## 4 Results

### 4.1 General Characteristics of the Experimental Animals

Measured biometric parameters were normalized to tibia length and presented in Table 2. Significant differences were observed in body weight, heart weight, and left ventricle (plus septum) weight of HanSD rats compared to TGR rats. Treatment with the sGC stimulator and ACEi did not affect the morphometric parameters in TGR rats. The untreated TGR ACF group was omitted from the table, since no rats survived until the end of the experiment.

**TABLE 2:** General characteristics of the experimental animals.

	TGR (*n* = 8)	TGR sGC (*n* = 9)	TGR ACF sGC (*n* = 2)	TGR ACF ACEi (*n* = 20)	TGR ACF sGC ACEi (*n* = 12)	HanSD (*n* = 9)
BW (g)	765 ± 13	770 ± 19	695 ± 43	723 ± 13	746 ± 25	549 ± 10*
HW/Tibia (mg/mm)	45.6 ± 1.2	41.7 ± 1.2	67.4 ± 0.3	65.6 ± 2.4	58.0 ± 3.5	33.7 ± 0.7*
LVW + S/Tibia (mg/mm)	34.5 ± 2.6	30.1 ± 2.5	46.2 ± 0.9	40.1 ± 4.6	37.1 ± 6.2	24.0 ± 1.4*
RVW/Tibia (mg/mm)	7.0 ± 0.8	7.5 ± 0.7	13.4 ± 1.4	15.3 ± 3.2	13.1 ± 3.5	6.5 ± 0.7
LAW/Tibia (mg/mm)	1.2 ± 0.4	1.2 ± 0.2	2.1 ± 0.1	2.2 ± 0.6	2.1 ± 0.8	0.8 ± 0.1
RAW/Tibia (mg/mm)	1.0 ± 0.2	1.1 ± 0.3	3.3 ± 0.1	4.4 ± 2.1	3.7 ± 1.8	1.0 ± 0.2

*Note:* Biometric parameters of the male normotensive sham HanSD rats, and hypertensive heterozygous Ren‐2 transgenic rats (TGR) with aortocaval fistula (ACF) or without ACF (sham), treated with sGC stimulator (BAY41‐8543), angiotensin‐converting enzyme inhibitor (ACEi), or their combination. Values are presented as mean ± SD; by one‐way ANOVA and Tukey's multiple comparison test. Number of experimental animals = number of rats that survived until the end of the 210‐day observation period.

Abbreviations: BW: body weight; HW: heart weight; LAW: left atrium weight; LVW + S: left ventricle plus septum weight; RAW: right atrium weight; RVW: right ventricle weight.

* *p* ≤ 0.05 vs. TGR.

### 4.2 Survival Rate After Long‐Term Treatment With sGC Stimulator and ACEi

The survival proportion of experimental animals is presented in Table S1. All of the HanSD rats survived until the end of the experiment (i.e., the end of the 210‐day observation period).

The untreated TGR rats with volume overload caused by ACF began to die rapidly in the first weeks of observation. Treatment with the sGC stimulator led to a significant improvement in the survival rate in comparison to the untreated group (TGR ACF). After 60 days of treatment, the survival rate was 50% compared to 8% in untreated animals. However, the effectiveness of the treatment started to fade out after day 140. No rats in this group survived until the end of the experiment. The best efficacy in decreasing mortality was achieved with ACEi administered alone (TGR ACF ACEi). The survival proportion in this group was 91% at the end of the observation period [20].

### 4.3 Nano‐Liquid Chromatography and Mass Spectrometry‐Based Proteomics in Left Ventricle Tissue

In the proteomic analyses measured in the left ventricular tissue, we focused on the proteins that were most significantly affected from the total of 179 identified proteins across all observed groups, as listed in Table S2. We selected proteins involved mainly in structural remodeling and oxidative damage/antioxidant defense (Table 3). Individual protein expression (upregulation or downregulation) is presented as ratios of protein abundances of two groups and referred to as a fold change.

**TABLE 3:** Identified proteins involved in the structural and oxidative remodeling.

Proteins	Abbreviation	TGR sGC vs. TGR		TGR vs. HanSD	
		Fold change	*p*	Fold change	*p*
Acylphosphatase‐2	ACYP2	0.949	0.785	1.295	0.101
Complement C3	CO3	1.154	0.084	1.102	0.294
Alpha‐crystallin B chain	CRYAB	1.164	0.381	0.980	0.872
Creatine kinase S‐type, mitochondrial	KCRS	1.214	0.066	0.634	**0.00002**
Vinculin	VINC	0.637	**0.003**	1.824	**0.0002**
Long‐chain‐fatty‐acid‐CoA ligase 1	ACSL1	1.487	0.057	1.076	0.777
Branched‐chain‐amino‐acid aminotransferase, mitochondrial	BCAT2	1.687	**0.014**	0.698	**0.038**
Destrin	DEST	0.790	0.195	1.120	0.491
Ig gamma‐2A chain C region	IGG2A	0.921	0.170	1.753	**0.000001**
Myosin‐binding protein C, cardiac‐type	MYPC	2.974	**0.000001**	1.461	0.052
NADH dehydrogenase [ubiquinone] flavoprotein 2, mitochondrial	NDUV2	1.664	**0.001**	0.592	**0.002**
PDZ and LIM domain protein 5	PDLI5	0.558	**0.001**	1.155	0.307
Bifunctional purine biosynthesis protein ATIC	PUR9	0.752	0.212	1.193	0.388
Cytochrome c, somatic	CYC	1.071	0.753	0.776	0.155
Transgelin‐2	TAGL2	1.328	0.101	1.077	0.665
Medium‐chain acyl‐CoA ligase ACSF2, mitochondrial	ACSF2	1.345	**0.011**	0.581	**0.0005**
10 kDa heat shock protein, mitochondrial	CH10	1.251	**0.038**	1.113	0.305
NADH–ubiquinone oxidoreductase 75 kDa subunit, mitochondrial	NDUS1	1.519	**0.001**	0.763	**0.014**
Superoxide dismutase [Cu‐Zn]	SODC	1.078	0.727	0.605	**0.029**
Amine oxidase [flavin‐containing] A	AOFA	0.878	0.482	3.118	**0.00004**
Atypical kinase COQ8A, mitochondrial	COQ8A	1.297	0.173	0.628	**0.020**
Carnitine O‐palmitoyltransferase 2, mitochondrial	CPT2	1.317	0.215	0.904	0.675
Glutamate dehydrogenase 1, mitochondrial	DHE3	1.644	**0.010**	0.495	**0.0003**
Glutathione peroxidase 1	GPX1	1.076	0.713	1.395	0.060
Glutathione S‐transferase Mu 2	GSTM2	1.322	**0.046**	0.621	**0.005**
Heat shock protein beta‐6	HSPB6	0.841	0.214	1.014	0.931
Propionyl‐CoA carboxylase alpha chain, mitochondrial	PCCA	1.585	**0.016**	0.459	**0.0001**
Prohibitin‐2	PHB2	1.115	0.700	1.199	0.507
Selenium‐binding protein 1	SBP1	0.921	0.694	0.878	0.519

*Note:* Selected proteins involved mainly in structural remodeling, oxidative damage/antioxidant defense measured by LC–MS in the left ventricular tissue of the hypertensive heterozygous Ren‐2 transgenic rats (TGR) with the sGC stimulator treatment or without the sGC stimulator (sham) and normotensive sham HanSD rats. Values in bold indicate significant changes (*p* ≤ 0.05). Values were considered statistically significant *p* < 0.05. Fold change values > 1 indicate a significant increase, while values < 1 indicate a significant decrease.

From identified proteins involved in the structural remodeling, protein levels of NADH dehydrogenase [ubiquinone] flavoprotein 2 (NDUV2), Branched‐chain amino acid aminotransferase (BCAT2), and Creatine kinase S‐type were significantly decreased, and Vinculin (VINC) as well as Ig gamma‐2A chain C region were upregulated in hypertensive TGR rats. Treatment with the sGC stimulator BAY41‐8543 normalized levels of NDUV2, BCAT2, and VINC in hypertensive TGR rats. Besides that, the sGC stimulator increased protein expression of Myosin‐binding protein C and decreased PDZ and LIM domain protein 5 in TGR rats.

The most significant changes in protein levels in relationship with oxidative damage due to hypertension in TGR rats were observed for amine oxidase [flavin‐containing] A (AOFA) protein and NADH–ubiquinone oxidoreductase 75 kDa subunit (NDUS1). Protein expression of AOFA was significantly elevated and NDUS1 was reduced in the heart tissue of TGR rats. On the other hand, proteins implicated in direct or indirect antioxidant defense systems such as Superoxide dismutase [Cu‐Zn], Glutamate dehydrogenase 1 (DHE3), Glutathione S‐transferase Mu 2 (GSTM2), Propionyl‐CoA carboxylase alpha chain (PCCA), Medium‐chain acyl‐CoA ligase (ACSF2), and atypical kinase COQ8A were significantly decreased in hypertensive TGR rats. Administration of sGC stimulator led to elevation of 10 kDa heat shock protein (CH10), ACSF2, NDUS1, DHE3, GSTM2, and PCCA in TGR rats, suggesting that these changes are involved in protection against oxidative damage.

### 4.4 Protein Levels Assessed by Western Blot Method in the Left Ventricle Tissue

#### 4.4.1 Proteins Related to Oxidative Stress/Antioxidant Defense and Inflammation

**FIGURE 2:**
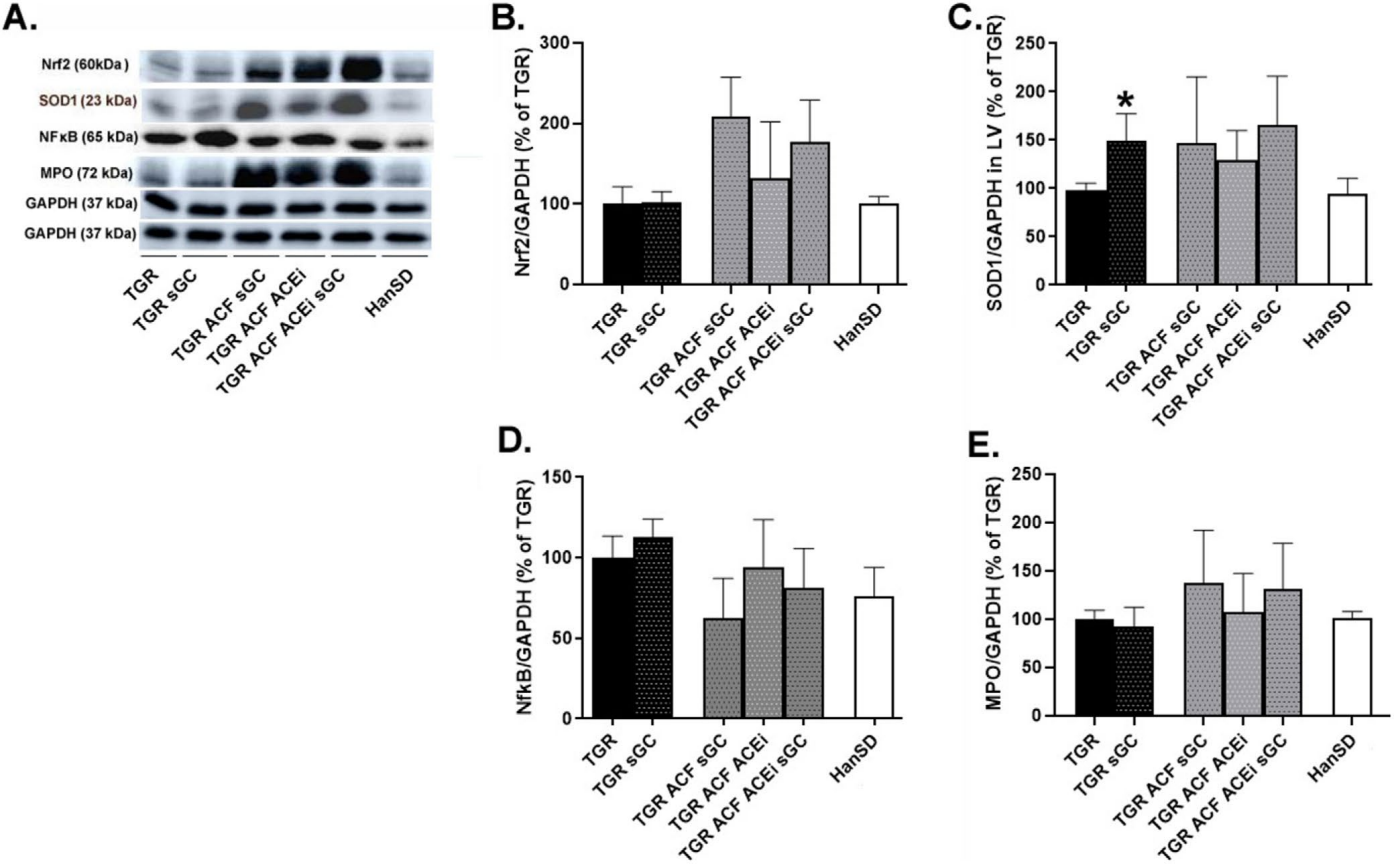
Myocardial protein levels and representative western blot images (A) of Nrf 2 (B), and antioxidant enzyme SOD1 (C) implicated in anti‐oxidant and anti‐inflammatory response, as well as NfκB (D) and MPO (E) related to inflammation and oxidative damage. Proteins were measured in the left ventricle of HanSD rat, and TGR rats with ACF or without (sham) that survived until the end of the treatment with sGC stimulator, ACEi or their combination. Values are normalized to GAPDH, presented as mean ± SD, *p ≤ 0.05 vs. TGR; by one‐way ANOVA and Tukey's multiple comparison test. Nrf2: Nuclear factor erythroid 2–related factor 2; SOD1: Superoxide dismutase 1; NfκB: Nuclear factor kappa B; MPO: Myeloperoxidase; GAPDH: Glyceraldehyde‐3‐phosphate dehydrogenase. Membranes for representative western blot images were cut and stripped for reprobing, which is the reason why only two GAPDH are shown.

The levels of proteins specific to anti‐oxidant and anti‐inflammatory defense, including nuclear factor erythroid 2–related factor (Nrf2) and superoxide dismutase 1 (SOD1), did not significantly change in TGR rats compared to HanSD. Treatment with the sGC stimulator (BAY41‐8543) had no significant effect on protein levels of Nrf2 in the TGR. However, it led to a significant increase in SOD1 levels in the TGR sham rats (Figure 2A–C).

The levels of proteins activated in response to stress including nuclear factor kappa B (NfκB) and myeloperoxidase (MPO) showed no significant changes in TGR rats compared to HanSD, although a tendency for increased NfκB protein levels was observed in TGR rats. Administration of the sGC stimulator (BAY41‐8543) had no significant effect on levels of NfκB and MPO in the TGR, as it is shown in Figure 2A,D,E.

#### 4.4.2 Proteins Related to Structural Remodeling

**FIGURE 3:**
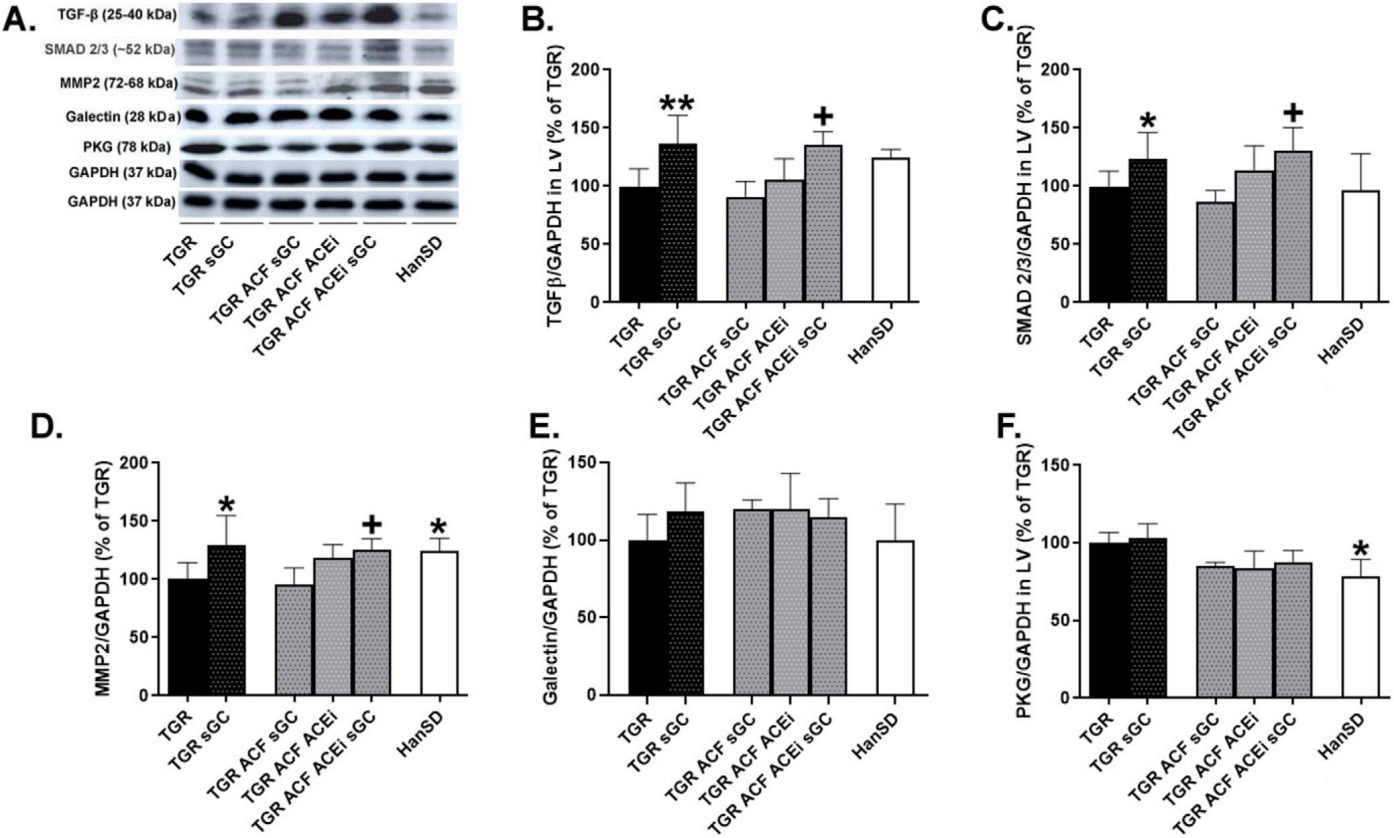
Myocardial protein levels and representative western blot images (A) of TGFβ (B), SMAD 2/3 (C), MMP2 (D), Galectin (E), and PKG (F) implicated in remodeling of the extracellular matrix, measured in the left ventricle of HanSD rats and TGR rats with ACF or without (sham) that survived until the end of the treatment with sGC stimulator, ACEi, or their combination. Values are normalized to GAPDH, presented as mean ± SD; *p ≤ 0.05 vs. TGR; ** *p* ≤ 0.005; + *p* ≤ 0.05 TGR ACF sGC vs. TGR ACF sGC ACEi, by one‐way ANOVA and Tukey's multiple comparison test. TGFβ: Transforming growth factor beta; SMAD 2/3: Mothers against decapentaplegic homolog 2/3; MMP2: Matrix metalloproteinase‐2; PKG: CGMP‐dependent protein kinase; GAPDH: Glyceraldehyde‐3‐phosphate dehydrogenase. Membranes for representative western blot images were cut and stripped for reprobing, which is the reason why only two GAPDH are shown.

The levels of proteins involved in extracellular matrix (ECM) remodeling were unchanged in TGR rats compared to HanSD (Figure 3), the cGMP‐dependent protein kinase (PKG) was significantly elevated in TGR rats (Figure 3A,F).

The protein levels of transforming growth factor beta (TGFβ), mothers against decapentaplegic homolog 2/3 (SMAD 2/3) and matrix metalloproteinase‐2 (MMP2) (Figure 3A–D) were significantly increased in the TGR sGC group compared to TGR sham rats. In the TGR ACF groups, levels of these proteins were significantly highest in the TGR ACF ACEi sGC rats.

Treatment with the sGC stimulator had no effect on levels of Galectin and PKG in the TGR group (Figure 3A,E).

#### 4.4.3 Proteins Involved in Apoptotic Signaling Pathways

**FIGURE 4:**
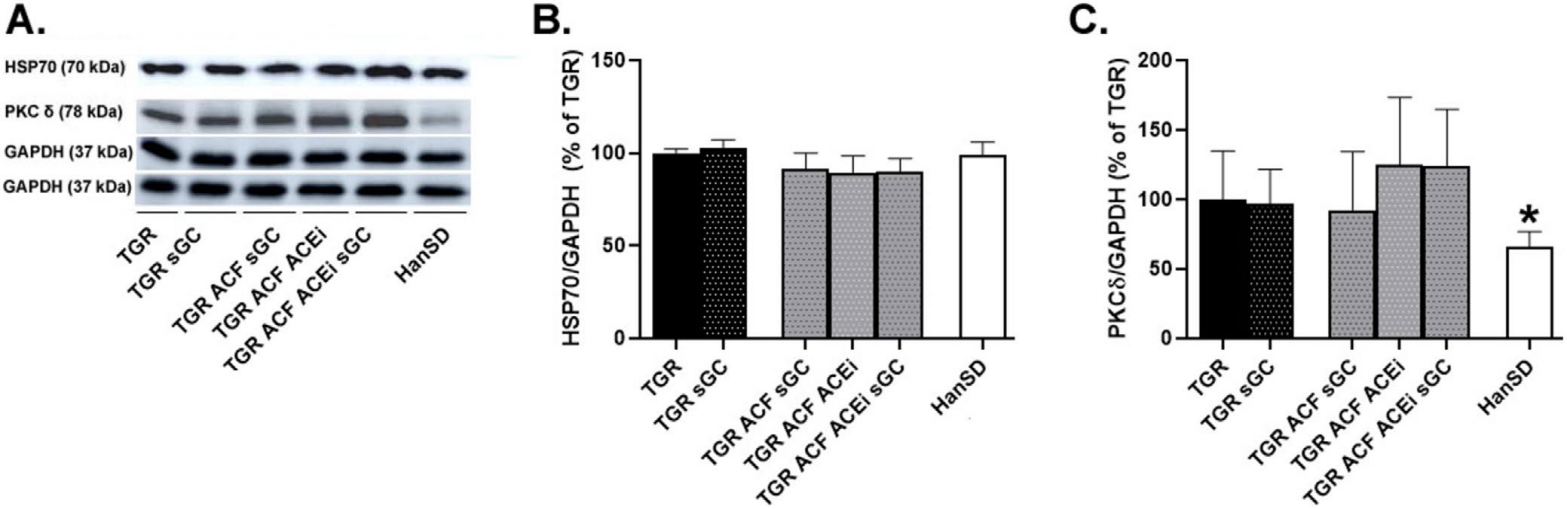
Myocardial protein levels and representative western blot images (A) of HSP70 (B) and PKCδ (C) implicated in apoptotic pathways, measured in the left ventricle of HanSD rats and TGR rats with ACF or without (sham) that survived until the end of the treatment with sGC stimulator, ACEi, or their combination. Values are normalized to GAPDH, presented as mean ± SD. *p ≤ 0.05 vs. TGR; by one‐way ANOVA and Tukey's multiple comparison test. HSP70: Heat shock protein 70; PKCδ: Protein kinase C delta; GAPDH: Glyceraldehyde‐3‐phosphate dehydrogenase. Membranes for representative western blot images were cut and stripped for reprobing, which is the reason why only two GAPDH are shown.

The protein levels of pro‐apoptotic/pro‐hypertrophic protein kinase C delta (PKCδ) were significantly upregulated in TGR rats compared to HanSD rats. We did not observe any effect of sGC stimulator on levels of HSP70 or PKCδ in the TGR group (Figure 4).

### 4.5 Histology and Enzyme Histochemistry of Left Ventricular Tissue

**FIGURE 5:**
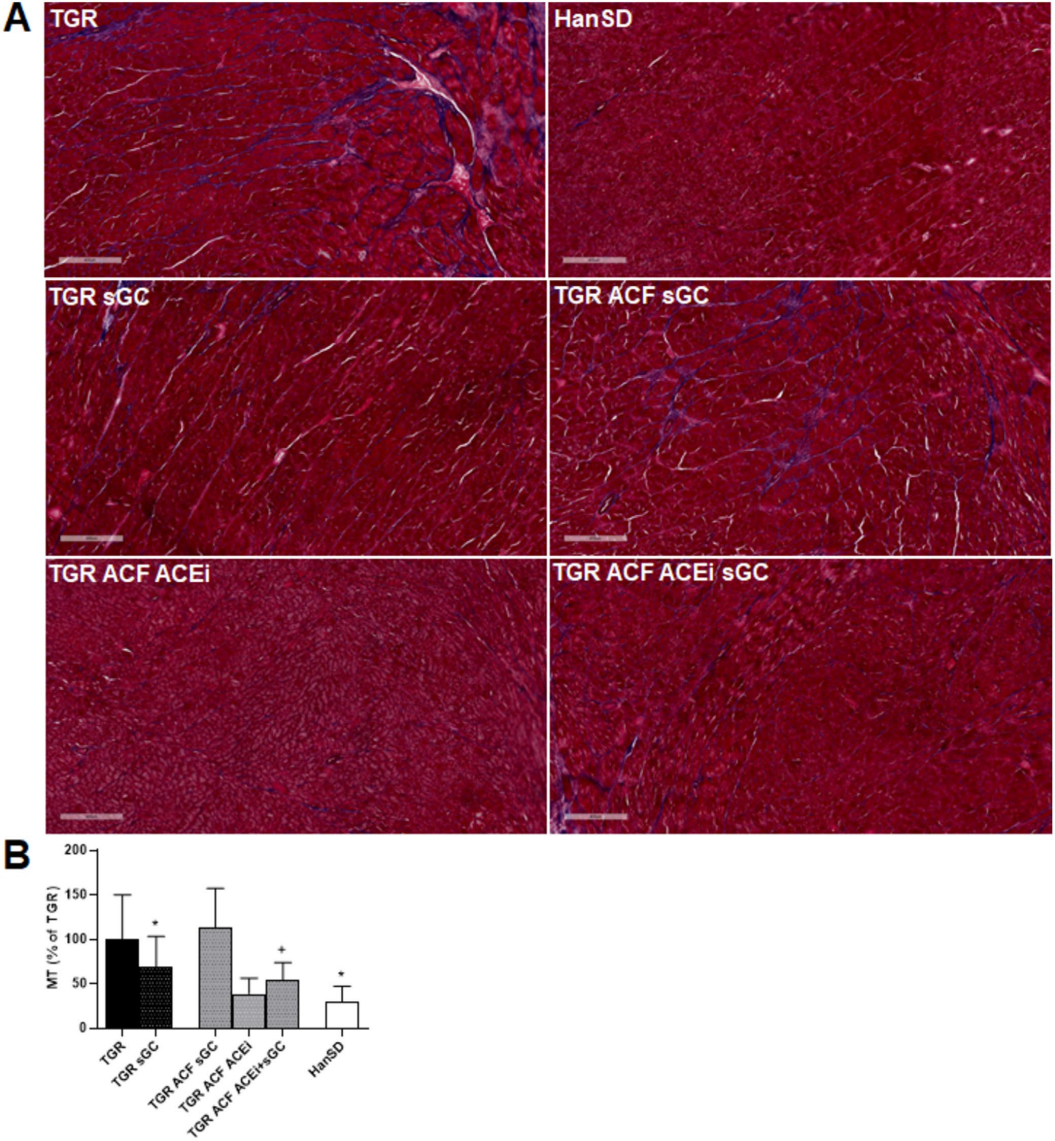
Masson's trichrome (MT) staining (A) and quantification of collagen deposition (B) in the left ventricle of HanSD rats, and TGR rats with ACF or without (sham) that survived until the end of the treatment with sGC stimulator, ACEi or their combination. Scale bar represents 400 μm. Values are presented as mean ± SD. *p ≤ 0.05 vs. TGR; + *p* ≤ 0.05 TGR ACF sGC vs. TGR ACF sGC ACEi; by one‐way ANOVA and Tukey's multiple comparison test.

For collagen detection (blue color), a conventional histological Masson's trichrome staining was performed. According to quantitative analysis, collagen deposition was highly increased in TGR rats compared to HanSD, and administration of the sGC stimulator led to a significant decrease in collagen deposition in TGR rats. We have also observed a significantly lower deposition of collagen in the TGR ACF ACEi + sGC group compared to the TGR ACF+ sGC group of rats, shown in Figure 5.

**FIGURE 6:**
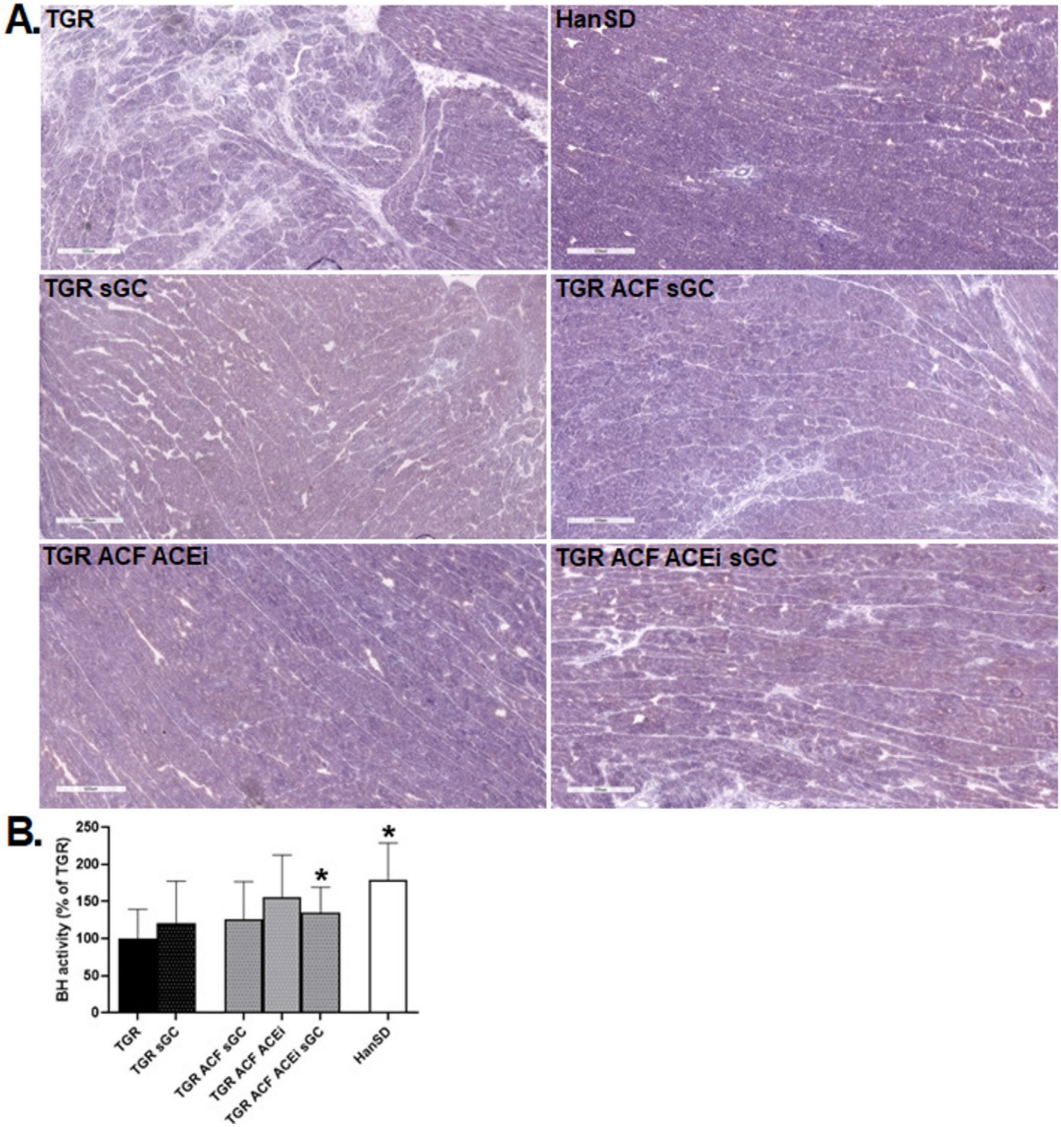
Histochemical detection of β‐hydroxybutyrate dehydrogenase activity—a marker of the intensity of β‐oxidation of fatty acids (A), and quantification of β‐hydroxybutyrate dehydrogenase activity (B) in the left ventricle of HanSD rat, and TGR rats with ACF or without (sham) that survived until the end of the treatment with sGC stimulator, ACEi or their combination. Scale bar represents 500 μm. Values are presented as mean ± SD. *p ≤ 0.05 vs. TGR; by one‐way ANOVA and Tukey's multiple comparison test.

Histochemical reaction of β‐hydroxybutyrate dehydrogenase (BHD) showed impaired and decreased BHD activity in TGR rats and rats with volume overload compared to HanSD. Treatment with the sGC stimulator separately did not indicate any significant changes in the activity of BHD, only in combination with ACEi in TGR ACF rats compared to TGR rats (Figure 6).

## 5 Discussion

In our previous study, we showed that the sGC stimulator (BAY41‐8543) improved the survival rate of hypertensive TGR rats with HF due to the volume overload (ACF); however, the possible mechanisms of this action were not fully elucidated [20]. The current study therefore aimed to investigate the efficacy of BAY 41–8543 on oxidative damage and adverse structural remodeling in the heart tissue in the same rats model of HF, which may fill in the missing information on the possible mechanism for mortality reduction.

Investigation of sGC stimulators as a treatment for heart disease has been under investigation for two decades, most recently for the treatment of HF since the sGC‐NO‐cGMP pathway is often disrupted under this pathology [28, 29, 30, 31]. The sGC stimulator, BAY 41–8543, used in the current study, has an equivalent mechanism to vericiguat [32], a drug administered mainly to HF patients with worsening symptoms [33, 34].

We used TGR rats as experimental animals, which represent a model of ANG II‐dependent hypertension with endogenous RAAS activation [35]. In addition, aortocaval fistula (ACF) was surgically created in TGR rats [19], which subsequently caused volume overload, RAAS activation, congestion, cardiac remodeling, and renal injury. The combination of these deleterious factors provides us with a suitable model to study HF [36].

Dose selection of BAY 41–8543, as well as the effect of the sGC stimulator on survival rate, was performed in our previous study [20]. Unfortunately, in the untreated TGR group with volume overload, none of the rats survived until the end of the experiment. In TGR rats with ACF treated with the sGC stimulator, the survival proportion was at least 7.1%, which is still a very low percentage compared to rats treated with angiotensin‐converting enzyme inhibitors (ACEi), where the survival rate was as high as 83%. ACEi, commonly used as a first‐line and most effective treatment for HF [37], served as a positive control treatment in this experiment. To further investigate the potential mechanisms implicated in survival improvement, we analyzed left ventricular samples using mass spectrometry, which offered us a wide range of insights into various pathways and their alterations.

Among the 179 measured proteins (Table S2), the most significantly altered proteins were those involved in oxidative damage, antioxidant protection, and structural remodeling (Table 3). Oxidative damage [38], as well as structural remodeling [39], are both interdependent courses of action that play key roles in the development and progression of HF [40].

From the first group of proteins affecting the ECM matrix (measured by mass spectrometry), the membrane‐cytoskeletal protein Vinculin was markedly decreased in TGR rats. Vinculin is one of the major components of the linkage system [41], and its protein levels are usually increased in patients with failing hearts, most probably as a compensatory mechanism for the loss of contractile filaments contributing to increased cardiomyocytes stiffness [42]. Treatment by sGC stimulator normalized Vinculin in hypertensive TGR rats. Additionally, it affected proteins such as Myosin‐binding protein C, NADH dehydrogenase [ubiquinone] flavoprotein 2, and PDZ and LIM domain protein 5. Impairment of these proteins promotes sarcomeric and mitochondrial dysfunction, as well as pathogenic mechanotransduction [43, 44, 45]. These results suggest that the sGC stimulator could improve cardiomyocyte contractile function, reduce mechanical stress, and so indirectly mitigate maladaptive ECM remodeling. However, this is in contradiction with increased protein levels of branched‐chain‐amino‐acid aminotransferase (BCAA) in TGR rats treated with sGC stimulator. BCAA is involved in cardiac energy homeostasis and in regulation of the mTOR signaling pathway implicated in fibroblast activation and fibrosis development, leading to increased stiffness [46, 47, 48]. In patients with HF, the levels of BCAA are mostly elevated [49].

Using the western blot method, we therefore focused specifically on proteins directly involved in fibroblast activation, synthesis of ECM proteins, degradation of ECM components, or facilitation of ECM turnover, such as TGF‐β‐SMAD [50], or matrix metalloproteinase proteins/enzymes [51]. The levels of these proteins were significantly elevated in TGR rats and TGR rats with ACF supplemented with sGC stimulator. Protein abundance of cardiac galectin, as a marker of inflammation and fibrosis [52], showed an increasing tendency in TGR rats treated with sGC stimulator.

Even more interesting was the finding that collagen deposition, as demonstrated by Masson's trichrome staining, was decreased in treated TGR and TGR ACF rats with sGC stimulator in combination with ACEi. Our previous study also reported a reduced tendency for collagen deposition after sGC supplementation in TGR and TGR ACF rats [20].

This dual role of sGC stimulator on cardiac structural remodeling is difficult to explain. One possible explanation is that the increased TGF‐β/SMAD signaling could be a compensatory response to ECM degradation, aiming to reload ECM components as MMP cleaves collagen. However, this is just a hypothesis, and further experiments are necessary to elucidate this issue.

From the second group of proteins related to oxidative damage, measured by mass spectrometry, the most markedly affected were: amine oxidase [flavin‐containing] A (AOFA) protein and NADH–ubiquinone oxidoreductase 75 kDa subunit (NDUS1). Protein expression of AOFA was significantly elevated and NDUS1 reduced in the heart tissue of TGR rats. The elevated protein levels of AOFA [53, 54] and decreased protein abundance of NDUS1 [55] have also been demonstrated in various animal models with HF. On the other hand, proteins implicated in direct or indirect antioxidant defense systems Superoxide dismutase [Cu‐Zn], Glutamate dehydrogenase 1, Glutathione S‐transferase Mu 2, Propionyl‐CoA carboxylase alpha chain, Medium‐chain acyl‐CoA ligase, Atypical kinase (COQ8A) were significantly decreased in hypertensive TGR rats. Downregulation of antioxidant enzymes is typical in the failing heart, suggesting reduced scavenging of ROS/RNS [56, 57]. Administration of the sGC stimulator led to normalization of NDUS1 and to elevation of some antioxidant enzymes in TGR rats, reflecting a partial protection against oxidative damage. Interestingly, AOFA, as one of the most regulated proteins in conditions of HF [53], was not affected by the sGC stimulator in hypertensive TGR rats. These results suggest that while the sGC stimulator exhibits some antioxidant activity, it does not possess the full antioxidant efficacy.

Results from western blot method concerning oxidative stress/antioxidant defense did not show any significant changes between groups in selected proteins, only increasing tendency of antioxidant enzyme superoxide dismutase after the treatment with sGC stimulator in TGR rats, what is in line with the results from mass spectrometry.

One of the characteristics of the failing human heart is a significant change in its energy metabolism. Therefore, it is also worth mentioning the results of histochemical detection of β‐hydroxybutyrate dehydrogenase (BDH) activity, an enzyme involved in ketone body metabolism. In conditions of chronic pathological cardiac overload, in failing hearts, especially when other energy sources are limited, ketone bodies are considered an alternative “fuel source” in myocardial energy metabolism [58, 59]. Our results showed the reduced BDH activity in TGR rats, as well as in TGR rats with volume overload, which indicates an energetically depleted myocardium with increased ketone utilization, where even alternative energy production is no longer sufficient. In addition, ketone bodies can regulate ROS production, reversing ROS‐induced DNA damage [25], which is also consistent with our results, where we showed that TGR rats suffered from significant cardiac oxidative stress.

sGC stimulator administered alone did not improve the ketone metabolism, which was improved only slightly in TGR rats with ACF treated in combination with ACEi.

## 6 Conclusion

The main purpose of the current study was to examine the efficiency of the sGC stimulator (BAY 41–8543) on adverse cardiac structural remodeling in conditions of heart failure, which occurs progressively over several months. A 210‐day long‐term treatment with the sGC stimulator demonstrated positive anti‐oxidant potential but a negative effect on the TGF‐SMAD pathways implicated in ECM remodeling in TGR rats.

**FIGURE 7:**
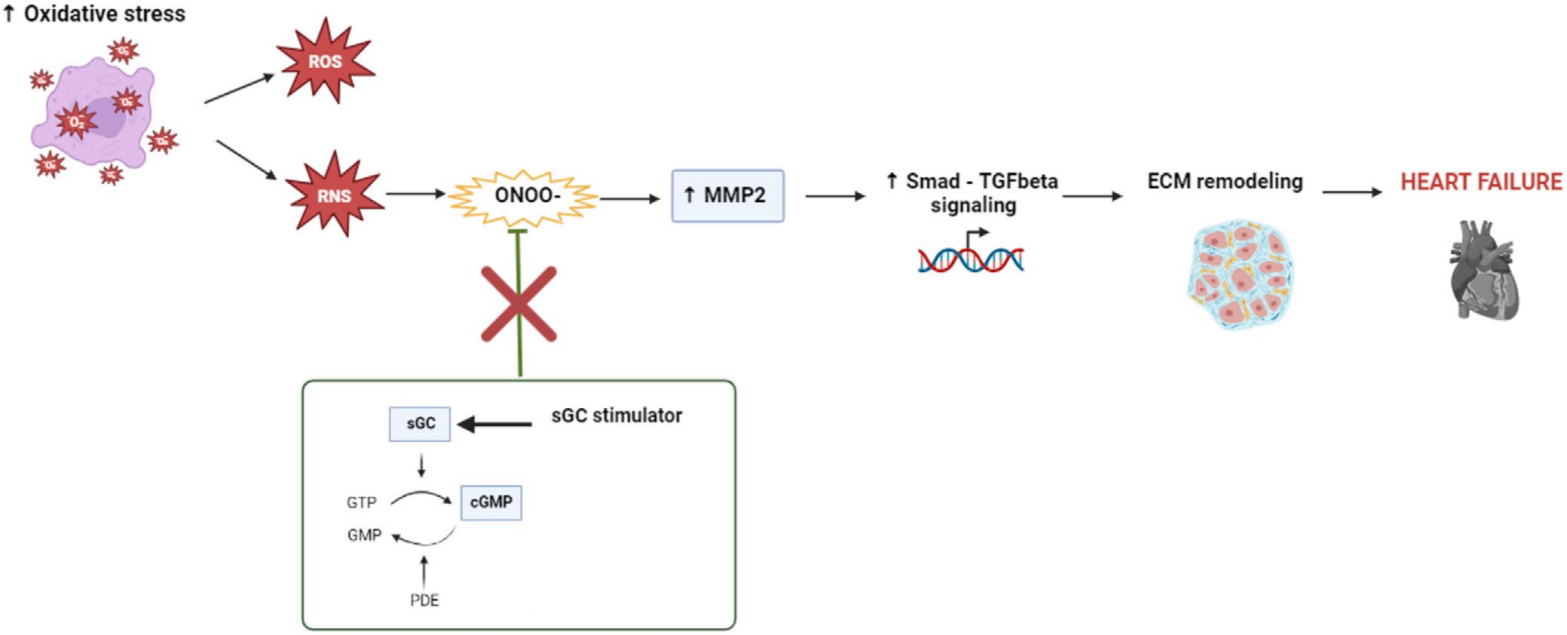
Schematic representation of a possible explanation of sGC stimulator adverse action on ECM remodeling. Oxidative stress was significantly increased in hypertensive TGR rats. Long‐term treatment with sGC stimulators normalized levels of ROS but presumably did not affect levels of Peroxynitrites (ONOO^−^) in hypertensive TGR rats. ONOO^−^ increases the activity of MMPs and subsequently enhances Smad‐TGF‐β signaling, promoting fibroblast activation and collagen synthesis (ECM remodeling), leading to impairment of heart function and heart failure. ROS/RNS: Reactive Oxygen/Nitrogen Species; TGFβ: transforming growth factor beta; SMAD 2/3: Mothers against decapentaplegic homolog 2/3; MMP2: matrix metalloproteinase‐2. (Created in BioRender.com).

We can assume that long‐term treatment with sGC stimulator reduced the levels of reactive oxygen species, but its effect on peroxynitrite (ONOO^−^) was less pronounced, which may explain the adverse effect of sGC stimulator on structural remodeling, since ONOO^−^ can directly modify ECM proteins (Figure 7).

Unfortunately, no rats survived in the untreated TGR group with volume overload, and therefore it was not possible to reliably assess the effect of the sGC stimulator in conditions of heart failure.

Based on our results, we can conclude that sGC stimulators could play an important role in the treatment of heart failure, probably in combination with other drugs, but more studies are needed to clarify the mechanisms of action.

## Author Contributions

**Adriana Martišková:** acquisition and analysis of data, writing – review and editing; **Matúš Sýkora:** acquisition and analysis of data, writing – review and editing; **Natália Andelová:** acquisition, analysis, and interpretation of data; **Miroslav Ferko:** acquisition and interpretation of data, funding acquisition; **Olga Gawrys:** project administration, conception and design of the study, writing – review and editing, funding acquisition; **Katarína Andelová:** acquisition and analysis of data, writing – review and editing; **Petr Kala:** conception and design of the study, interpretation of data; funding acquisition; **Luděk Červenka:** supervision, conception and design of the study, funding acquisition, writing – review and editing; **Barbara Szeiffová Bačová:** supervision; writing – original draft preparation, analysis and interpretation of data; funding acquisition. All authors have read and agreed to the published version of the manuscript.

## Disclosure

A major limitation of the study is the high mortality rate of TGR rats with induced volume overload by ACF formation. No rats survived to the end of the experiment in the untreated TGR rats with volume overload group, and only a few animals survived in the TGR ACF‐treated groups. Therefore, it was impossible to reliably assess the effect of the sGC stimulator in these groups of animals. We therefore excluded TGR ACF groups from the analyses in nano‐liquid chromatography and mass spectrometry‐based proteomics. In other analyses, the results from these groups are mostly demonstrative.

## Conflicts of Interest

The authors declare no conflicts of interest.

## Supporting information


Data S1.

## Data Availability

The data that support the findings of this study are in the Supporting Information material of this article and are available from the corresponding author upon reasonable request.
